# Supplementary material for: socialh: An R package for determining the social hierarchy of animals using data from individual electronic bins
Source: PLoS One. 2022 Aug 3;17(8):e0271337. doi: 10.1371/journal.pone.0271337 (PMC9348686; doi:10.1371/journal.pone.0271337)
Supplement: Attachment — Submitted filename: Response to Reviewers.docx [file pone.0271337.s001.docx]

fPONE-D-21-29427

**socialh: An R package for determining the social hierarchy of cattle using data from individual electronic bins**

Journal requirements:

1. When submitting your revision, we need you to address these additional requirements.

Please ensure that your manuscript meets PLOS ONE's style requirements, including those for file naming. The PLOS ONE style templates can be found at

[https://journals.plos.org/plosone/s/file?id=wjVg/PLOSOne_formatting_sample_main_body.pdf](https://na01.safelinks.protection.outlook.com/?url=https%3A%2F%2Fjournals.plos.org%2Fplosone%2Fs%2Ffile%3Fid%3DwjVg%2FPLOSOne_formatting_sample_main_body.pdf&data=04%7C01%7C%7Caa74d860d16c41f6cacc08d9be9e83c5%7C84df9e7fe9f640afb435aaaaaaaaaaaa%7C1%7C0%7C637750410304807027%7CUnknown%7CTWFpbGZsb3d8eyJWIjoiMC4wLjAwMDAiLCJQIjoiV2luMzIiLCJBTiI6Ik1haWwiLCJXVCI6Mn0%3D%7C3000&sdata=SZRFHU3skQloQiQTlXUy0yraZQtZL3sbpp%2B19UlBJYo%3D&reserved=0) and

[https://journals.plos.org/plosone/s/file?id=ba62/PLOSOne_formatting_sample_title_authors_affiliations.pdf](https://na01.safelinks.protection.outlook.com/?url=https%3A%2F%2Fjournals.plos.org%2Fplosone%2Fs%2Ffile%3Fid%3Dba62%2FPLOSOne_formatting_sample_title_authors_affiliations.pdf&data=04%7C01%7C%7Caa74d860d16c41f6cacc08d9be9e83c5%7C84df9e7fe9f640afb435aaaaaaaaaaaa%7C1%7C0%7C637750410304816989%7CUnknown%7CTWFpbGZsb3d8eyJWIjoiMC4wLjAwMDAiLCJQIjoiV2luMzIiLCJBTiI6Ik1haWwiLCJXVCI6Mn0%3D%7C3000&sdata=Q50obs5UNvLQuVgsVjbbXkzHgv7wQ%2F%2F%2FbJS7IbrslSo%3D&reserved=0)

AU: We reviewed the manuscript to fit the PLOS ONE's style requirements.

2. Thank you for stating the following financial disclosure:

“We thank the São Paulo Research Foundation (FAPESP -  [https://fapesp.br/en](https://na01.safelinks.protection.outlook.com/?url=https%3A%2F%2Ffapesp.br%2Fen&data=04%7C01%7C%7Caa74d860d16c41f6cacc08d9be9e83c5%7C84df9e7fe9f640afb435aaaaaaaaaaaa%7C1%7C0%7C637750410304816989%7CUnknown%7CTWFpbGZsb3d8eyJWIjoiMC4wLjAwMDAiLCJQIjoiV2luMzIiLCJBTiI6Ik1haWwiLCJXVCI6Mn0%3D%7C3000&sdata=htj%2F%2BZlCkHeLHYiHvIxA6J2mUFZWTu8odI7G7%2FHPlK4%3D&reserved=0); grant #2017/10630-2 and grant #2017/50339-5).

JPSV, MD, and KTS acknowledge the Coordination for the Improvement of High Education Personnel (CAPES - [https://www.iie.org/programs/capes](https://na01.safelinks.protection.outlook.com/?url=https%3A%2F%2Fwww.iie.org%2Fprograms%2Fcapes&data=04%7C01%7C%7Caa74d860d16c41f6cacc08d9be9e83c5%7C84df9e7fe9f640afb435aaaaaaaaaaaa%7C1%7C0%7C637750410304826947%7CUnknown%7CTWFpbGZsb3d8eyJWIjoiMC4wLjAwMDAiLCJQIjoiV2luMzIiLCJBTiI6Ik1haWwiLCJXVCI6Mn0%3D%7C3000&sdata=YdxVsgRqlfsXgYFJPEezcTAN74ZwYAFc0MLGNZlBWIA%3D&reserved=0) ) for the scholarship.”

Please state what role the funders took in the study.  If the funders had no role, please state: ""The funders had no role in study design, data collection and analysis, decision to publish, or preparation of the manuscript.""

If this statement is not correct you must amend it as needed.

Please include this amended Role of Funder statement in your cover letter; we will change the online submission form on your behalf.

AU: We corrected the fund information: “We thank the São Paulo Research Foundation (FAPESP -  [https://fapesp.br/en](https://na01.safelinks.protection.outlook.com/?url=https%3A%2F%2Ffapesp.br%2Fen&data=04%7C01%7C%7Caa74d860d16c41f6cacc08d9be9e83c5%7C84df9e7fe9f640afb435aaaaaaaaaaaa%7C1%7C0%7C637750410304816989%7CUnknown%7CTWFpbGZsb3d8eyJWIjoiMC4wLjAwMDAiLCJQIjoiV2luMzIiLCJBTiI6Ik1haWwiLCJXVCI6Mn0%3D%7C3000&sdata=htj%2F%2BZlCkHeLHYiHvIxA6J2mUFZWTu8odI7G7%2FHPlK4%3D&reserved=0); grant #2017/10630-2 and grant #2017/50339-5) for the financial support to perform data collection and to publish this article. The funders had no role in study design, data collection and analysis, decision to publish, or preparation of the manuscript.”

3.Thank you for stating the following in the Acknowledgments Section of your manuscript:

“We thank the São Paulo Research Foundation (FAPESP, grant #2017/10630-2 and grant #2017/50339-5). We thank the staffs of Beef Cattle Research Center, Institute of Animal Science, Sertãozinho, São Paulo State, Brazil for helping in the data acquisition. JPSV, MD, and KTS acknowledge CAPES (Coordination for the Improvement of High Education Personnel) for the scholarship. Finally, we also thank the professor Luiz Carlos Pinheiro Machado Filho, PhD for all the shared knowledge that motivated us to work with social behavior.”

We note that you have provided funding information that is not currently declared in your Funding Statement. However, funding information should not appear in the Acknowledgments section or other areas of your manuscript. We will only publish funding information present in the Funding Statement section of the online submission form.

Please remove any funding-related text from the manuscript and let us know how you would like to update your Funding Statement. Currently, your Funding Statement reads as follows:

“We thank the São Paulo Research Foundation (FAPESP -  [https://fapesp.br/en](https://na01.safelinks.protection.outlook.com/?url=https%3A%2F%2Ffapesp.br%2Fen&data=04%7C01%7C%7Caa74d860d16c41f6cacc08d9be9e83c5%7C84df9e7fe9f640afb435aaaaaaaaaaaa%7C1%7C0%7C637750410304826947%7CUnknown%7CTWFpbGZsb3d8eyJWIjoiMC4wLjAwMDAiLCJQIjoiV2luMzIiLCJBTiI6Ik1haWwiLCJXVCI6Mn0%3D%7C3000&sdata=kionfvtmCUf%2FlRBdzZWki5f7l%2BOzyS%2FG%2FPOfbIiO2yk%3D&reserved=0); grant #2017/10630-2 and grant #2017/50339-5).

JPSV, MD, and KTS acknowledge the Coordination for the Improvement of High Education Personnel (CAPES - [https://www.iie.org/programs/capes](https://na01.safelinks.protection.outlook.com/?url=https%3A%2F%2Fwww.iie.org%2Fprograms%2Fcapes&data=04%7C01%7C%7Caa74d860d16c41f6cacc08d9be9e83c5%7C84df9e7fe9f640afb435aaaaaaaaaaaa%7C1%7C0%7C637750410304836901%7CUnknown%7CTWFpbGZsb3d8eyJWIjoiMC4wLjAwMDAiLCJQIjoiV2luMzIiLCJBTiI6Ik1haWwiLCJXVCI6Mn0%3D%7C3000&sdata=6XvVlFNB4Wq6CjrXHk9kVN9YTBaaXssGrxQaTswSeZI%3D&reserved=0) ) for the scholarship.”

Please include your amended statements within your cover letter; we will change the online submission form on your behalf.

AU: Sorry for the mistake. We excluded the funding information from the acknowledgement section.

4. In your Data Journal Requirements: Availability statement, you have not specified where the minimal data set underlying the results described in your manuscript can be found. PLOS defines a study's minimal data set as the underlying data used to reach the conclusions drawn in the manuscript and any additional data required to replicate the reported study findings in their entirety. All PLOS journals require that the minimal data set be made fully available. For more information about our data policy, please see [http://journals.plos.org/plosone/s/data-availability](https://na01.safelinks.protection.outlook.com/?url=http%3A%2F%2Fjournals.plos.org%2Fplosone%2Fs%2Fdata-availability&data=04%7C01%7C%7Caa74d860d16c41f6cacc08d9be9e83c5%7C84df9e7fe9f640afb435aaaaaaaaaaaa%7C1%7C0%7C637750410304836901%7CUnknown%7CTWFpbGZsb3d8eyJWIjoiMC4wLjAwMDAiLCJQIjoiV2luMzIiLCJBTiI6Ik1haWwiLCJXVCI6Mn0%3D%7C3000&sdata=0hkjzWPqUWfXQHI112jqsqu%2BHfEB9q%2FE%2FuQl12qRvpw%3D&reserved=0).

Upon re-submitting your revised manuscript, please upload your study’s minimal underlying data set as either Supporting Information files or to a stable, public repository and include the relevant URLs, DOIs, or accession numbers within your revised cover letter. For a list of acceptable repositories, please see [http://journals.plos.org/plosone/s/data-availability#loc-recommended-repositories](https://na01.safelinks.protection.outlook.com/?url=http%3A%2F%2Fjournals.plos.org%2Fplosone%2Fs%2Fdata-availability%23loc-recommended-repositories&data=04%7C01%7C%7Caa74d860d16c41f6cacc08d9be9e83c5%7C84df9e7fe9f640afb435aaaaaaaaaaaa%7C1%7C0%7C637750410304846859%7CUnknown%7CTWFpbGZsb3d8eyJWIjoiMC4wLjAwMDAiLCJQIjoiV2luMzIiLCJBTiI6Ik1haWwiLCJXVCI6Mn0%3D%7C3000&sdata=5sOmL2kT2kv2Km%2BZ4WsTshvLGx3WpciEA%2BHFrGyshv0%3D&reserved=0). Any potentially identifying patient information must be fully anonymized.

Important: If there are ethical or legal restrictions to sharing your data publicly, please explain these restrictions in detail. Please see our guidelines for more information on what we consider unacceptable restrictions to publicly sharing data: [http://journals.plos.org/plosone/s/data-availability#loc-unacceptable-data-access-restrictions](https://na01.safelinks.protection.outlook.com/?url=http%3A%2F%2Fjournals.plos.org%2Fplosone%2Fs%2Fdata-availability%23loc-unacceptable-data-access-restrictions&data=04%7C01%7C%7Caa74d860d16c41f6cacc08d9be9e83c5%7C84df9e7fe9f640afb435aaaaaaaaaaaa%7C1%7C0%7C637750410304846859%7CUnknown%7CTWFpbGZsb3d8eyJWIjoiMC4wLjAwMDAiLCJQIjoiV2luMzIiLCJBTiI6Ik1haWwiLCJXVCI6Mn0%3D%7C3000&sdata=lT67DcYu6f0uFUQbkjFDMPbrFeaQFiXYdMZViI0N8OY%3D&reserved=0). Note that it is not acceptable for the authors to be the sole named individuals responsible for ensuring data access.

We will update your Data Availability statement to reflect the information you provide in your cover letter.

AU: The database used in the examples is available in socialh package (<https://cran.r-project.org/web/packages/socialh/index.html>) and Kaggle repository, (https://www.kaggle.com/datasets/juliavalente/data-from-visits-to-the-trough-of-nellore-cattle).

5. Your ethics statement should only appear in the Methods section of your manuscript. If your ethics statement is written in any section besides the Methods, please delete it from any other section.

AU: Done.

**Additional Editor Comments:**

Dear Authors,
The manuscript have certain suggestions from the reviewers that must be addressed before the final decision. Please respond to the comments of the reviewers and upload a revised version of your manuscript. Thank you.

[Note: HTML markup is below. Please do not edit.]

Reviewers' comments:

Reviewer's Responses to Questions

**Comments to the Author**

1. Is the manuscript technically sound, and do the data support the conclusions?
The manuscript must describe a technically sound piece of scientific research with data that supports the conclusions. Experiments must have been conducted rigorously, with appropriate controls, replication, and sample sizes. The conclusions must be drawn appropriately based on the data presented.

Reviewer #1: Partly

Reviewer #2: Partly

2. Has the statistical analysis been performed appropriately and rigorously?

Reviewer #1: N/A

Reviewer #2: N/A

3. Have the authors made all data underlying the findings in their manuscript fully available?
The [PLOS Data policy](https://na01.safelinks.protection.outlook.com/?url=http%3A%2F%2Fwww.plosone.org%2Fstatic%2Fpolicies.action%23sharing&data=04%7C01%7C%7Caa74d860d16c41f6cacc08d9be9e83c5%7C84df9e7fe9f640afb435aaaaaaaaaaaa%7C1%7C0%7C637750410304846859%7CUnknown%7CTWFpbGZsb3d8eyJWIjoiMC4wLjAwMDAiLCJQIjoiV2luMzIiLCJBTiI6Ik1haWwiLCJXVCI6Mn0%3D%7C3000&sdata=FyH%2Fk0yWJVteaMDdMZRiiMWB%2BZ89SYv1Zk2u3h0%2BGUc%3D&reserved=0) requires authors to make all data underlying the findings described in their manuscript fully available without restriction, with rare exception (please refer to the Data Availability Statement in the manuscript PDF file). The data should be provided as part of the manuscript or its supporting information or deposited to a public repository. For example, in addition to summary statistics, the data points behind means, medians and variance measures should be available. If there are restrictions on publicly sharing data—e.g. participant privacy or use of data from a third party—those must be specified.

Reviewer #1: No

Reviewer #2: No

4. Is the manuscript presented in an intelligible fashion and written in standard English?
PLOS ONE does not copyedit accepted manuscripts, so the language in submitted articles must be clear, correct, and unambiguous. Any typographical or grammatical errors should be corrected at revision, so please note any specific errors here.

Reviewer #1: Yes

Reviewer #2: Yes

5. Review Comments to the Author

Please use the space provided to explain your answers to the questions above. You may also include additional comments for the author, including concerns about dual publication, research ethics, or publication ethics. (Please upload your review as an attachment if it exceeds 20,000 characters)

**Reviewer #1:** The paper presents an interesting approach as an R package to evaluate social hierarchy. However, there are aspects of that could be improved. The example lacks on detail, which makes the reader hard to follow. In addition, the intervals (line 121) seem critical to evaluate the social hierarchy, and the references used are from Holstein dairy cows and the authors mentioned that there is an effect of the species and category, so what happened with the example and the intervals used- where no Holstein cows? what happens when there are more than 2 animals – no dyadic? I suggest adding a section with the limitations of the package. The text will benefit to have a better explanation of the references cited (lines 157-167), is very hard to follow the idea if there is not a little more elaboration of the cited literature.

AU: We rewrote some parts of the text to make it clear. In Ln135-149, we add an explanation for why we presented the references from Holstein dairy and apply the socialh package with data from Nellore cattle. We emphasized that our objective is not determine the optimal time-interval for identify replacement. Also, we make researchers free to adopt any time-interval.

Specific comments:
L25: Farm animal welfare instead of the welfare of farm animals.

AU: Done.

L30: creating instead of crated

AU: Done.

L55: Replacement need a definition

AU: We include a sentence of definition in Ln52-54: “A replacement occurs when an animal hat occupied the bin (reactor) is completely withdrawn by another animal that occupied the same bin (actor) in a short interval.”

L58: what does flexible means?

AU: It means that the socialh package can be applied in several studies, with different animals species and categories (young or old), and the user has two ways to import the dataset: (1) data from electronic bins; (2) data from animals’ observation. Also, due to the R software be open source, it is possible to the researcher add function from other packages.

Ln61-62: “Our package is intended for users who wish to implement more flexible (i.e. it is possible include data from electronics bins and data observational) social behaviour analysis since the R environment allows the integration of several functions from different packages.”

Table 1: what happens when there are more than 2 animals?

AU: For social behavior analysis, we always consider the disputes that occur between two animals (dyad), as it is necessary to identify the winning and losing animal. The disputes between two animals engaged in establishing and maintaining their mutual dominance-subordinate status include an array of experiences that can have positive or negative reinforcement of their actions, depending on the outcome of each interaction.

L 80: Bins

AU: Done.

L101-102: sentence is not clear

AU: We rewrote the sentence, and hope be clearest now.

Ln96-99: “The Total Mixed Ration (TMR) was offered twice a day (8:00 and 15:00 hours) and the animals had *ad libitum* access to TMR and water trough. All animals were fitted with an ear tag transponder that allowed the electronic feeding system to record the date and time when each animal entered and left the feeder.”

L115: defined – describing

AU: Done.

L125: This need to be clarified- there is an effect of species and category, but you run an example in Nellore cattle using intervals from Holstein?

AU: The main objective of this manuscript was to show the socialh package application and not validate the optimal time interval for identifying replacements of Nellore cattle. We used the Holstein reference, as they were the only literature available, to our knowledge, that used data from electronic bins to determine replacement. However, our present manuscript is part of a larger project that includes experiments with Nellore cattle. So, to test the package we used the data available from feed efficiency test of Nellore cattle available by the Institute of Animal Science. We included the explanation in Ln 143-149.

L157-167: the methods mentioned here need a brief explanation as least

AU: We included a brief explanation of the other methods. Ln 202-212.

L210: to the dominance

AU: Done.

L214: what does SH stands for?

AU: Sorry for the mistake. We include the definition of SH (social hierarchy) on Ln255.

**Reviewer #2:** The manuscript describes the existence and basic use of the 'socialh' R package.

In general, the manuscript is well written. However, there are many minor English language usage issues throughout the text. These could probably be remedied easily by having a native speaker proofread the text.

The software seems reasonable as a starting point. It is somewhat rudimentary in its implementation, however, and lacks the following basic pieces:

* Included sample data sets
* Package vignette
* Fleshed-out help functions
AU: Done

The package should be considered as alpha-level at this point.

In fact, the paper as it currently stands is really only at the level of an first-draft of a package vignette. It does not rise to the level needed to publish in PLOS ONE.

To make this paper publishable in PLOS ONE, I think that most of the following are needed:

* Demonstrate or add at least some capability to perform visualization of the results.

AU: Thank you for your suggestion. We add three functions to visualize the results obtained from the social package. See details in the section “Visualization of the results”. Ln 278-295.

* Demonstrate or add at least some capability to perform statistical analysis.

For example, either implement statistical theory around the Landau index or the improved index. This could be done without much effort using the bootstrap, probably, but perhaps there is actually some theoretical work around these statistics.
AU: Linearity indices were better explained in the text. See Ln 296-307.

* Perform at least three analyses of data that are novel in some way. This could be analyzing datasets that the authors have available, or data from other researchers interested.

* Or, alternatively, using data from the literature, perform analyses that add to the understanding of the data or that amplify, verify, or contradict the already published analyses.

AU: To exemplify the use of the socialh package, we used real data collected by electronic bins system in a feed efficiency test carried out at Institute of Animal Science, Sertãozinho, São Paulo, Brazil.

* Demonstrate the utility for large databases that is claimed in Line 285. A large database these days is probably on the order of 10 million records or more.

AU: We understand your concern. However, it is not common in behavior studies to obtain a large database of 10 million records or more. Also, performing an analysis of a large database requires a powerful computer.

To our knowledge, the R environment has no limitation regarding the length of the database, unlike other software like Excel.

* Optionally, add additional linearity evaluations and provide some empirical evaluation of them via simulation, followed by an evaluation of several real data sets.

AU: We thank you for your suggestion and we plan make this improvement for next socialh package update.

* Optionally, perform a set of simulations that adds to the ability of researchers to meaningfully choose among ranking, or provides some sort of empirical insight into the use of the software, or provides some empirical validation of the interpretation of the outputs.

For future work, it might be useful to consider the following:
* Generalize the use of methodology to determine dominance.
That is, allow the user to specify different methodologies for this step. The authors list several, but hard-code only one.

Flexibility could be achieved by using a function name as an input parameter, for example, where the function is expected to follow a specific API. That would allow a user to program their own methodology quite easily. This would avoid the authors' having to provide a hard-coded list of options. R provides other methods for increasing flexibility, this is only one suggestion.
This would allow demonstrating empirically that different interpretations using different dominance evaluations, for example.
AU: This is a good idea, and we can include other methodologies that have not yet been implemented in R to determine the dominance value of animals in the future. For now, the main objective was to make the package available based on the methodology of Kondo and Hurnik (1990), as there is no package that performs such method.

* Similarly, make social rank a user-definable set of cut-points, rather than hard-coded as only 3 values and 3 labels, and at the default cut-points provided by the R cut() function.

AU: The dvalue() function has been changed to [dvalue(dmatrix, rs = TRUE, hs = TRUE)], and two options of results visualization were added. Now the user can decide which outputs wants to obtain (social hierarchy and/or social rank). Ln 272.

As we are following the methodology proposed by Coimbra, Machado Filho & Hötzel (2012), it is not possibly made available to the user choose the cutoff point. Nevertheless, the user can use the complete list of dominance value of each animal to perform the cutoff point according to it research objective.

At a minimum, here, define these in the start of the function.

* In general, avoid any hard-coding of parameters inside the functions.
Make these user-definable at best or define them using variables at the start of the function at worst. At least the latter prepares the code for future generalization and makes coding decisions more visible.

AU: We verified the functions provided in the package to reduce the hard code; however, the functions are based on methodologies available in the literature (Coimbra, Machado Filho & Hötzel, 2012; Kondo & Hurnik, 1995), so the parameters are already defined.

* If visualization methods are constructed, add ggplot interfaces so that users can automatically have access to a flexible graphical presentation system.

AU: We add three functions to visualize the results obtained from the socialh package. See details in the section “Visualization of the results”. Ln 278-295.

6. PLOS authors have the option to publish the peer review history of their article ([what does this mean?](https://na01.safelinks.protection.outlook.com/?url=https%3A%2F%2Fjournals.plos.org%2Fplosone%2Fs%2Feditorial-and-peer-review-process%23loc-peer-review-history&data=04%7C01%7C%7Caa74d860d16c41f6cacc08d9be9e83c5%7C84df9e7fe9f640afb435aaaaaaaaaaaa%7C1%7C0%7C637750410304856807%7CUnknown%7CTWFpbGZsb3d8eyJWIjoiMC4wLjAwMDAiLCJQIjoiV2luMzIiLCJBTiI6Ik1haWwiLCJXVCI6Mn0%3D%7C3000&sdata=bocvKRA7uhTdIvSUN7Y8qAp%2Fx%2FNjGShdL1Thsd3HCGo%3D&reserved=0)). If published, this will include your full peer review and any attached files.

If you choose “no”, your identity will remain anonymous but your review may still be made public.


**Do you want your identity to be public for this peer review?** For information about this choice, including consent withdrawal, please see our [Privacy Policy](https://na01.safelinks.protection.outlook.com/?url=https%3A%2F%2Fwww.plos.org%2Fprivacy-policy&data=04%7C01%7C%7Caa74d860d16c41f6cacc08d9be9e83c5%7C84df9e7fe9f640afb435aaaaaaaaaaaa%7C1%7C0%7C637750410304856807%7CUnknown%7CTWFpbGZsb3d8eyJWIjoiMC4wLjAwMDAiLCJQIjoiV2luMzIiLCJBTiI6Ik1haWwiLCJXVCI6Mn0%3D%7C3000&sdata=mFdnKyDwyglBxiyZNJEdoEoRXnbrvu%2B9gtAmakgDkIc%3D&reserved=0).

Reviewer #1: No

Reviewer #2: No
