# Supplementary material for: socialh: An R package for determining the social hierarchy of animals using data from individual electronic bins
Source: PLoS One. 2022 Aug 3;17(8):e0271337. doi: 10.1371/journal.pone.0271337 (PMC9348686; doi:10.1371/journal.pone.0271337)
Supplement: Attachment — Submitted filename: Response to Reviewers.docx [file pone.0271337.s002.docx]

**PONE-D-21-29427R1**

**socialh : An R package for determining the social hierarchy of animals using data from individual electronic bins**

4. Have the authors made all data underlying the findings in their manuscript fully available?

The PLOS Data policy requires authors to make all data underlying the findings described in their manuscript fully available without restriction, with rare exception (please refer to the Data Availability Statement in the manuscript PDF file). The data should be provided as part of the manuscript or its supporting information, or deposited to a public repository. For example, in addition to summary statistics, the data points behind means, medians and variance measures should be available. If there are restrictions on publicly sharing data—e.g. participant privacy or use of data from a third party—those must be specified.

Reviewer #1: Yes

Reviewer #2: No

Answer: Ln 92-94: “The database used in the examples is available on socialh package and Kaggle repository (https://www.kaggle.com/datasets/juliavalente/data-from-visits-to-the-trough-of-nellore-cattle)”.

6. Review Comments to the Author

Please use the space provided to explain your answers to the questions above. You may also include additional comments for the author, including concerns about dual publication, research ethics, or publication ethics. (Please upload your review as an attachment if it exceeds 20,000 characters)

Reviewer #1: The authors have addressed all the comments and with that the paper has improve and therefore I considered that can be publish.

Reviewer #2: Although the authors have made some additions to their manuscript, the overall level of the manuscript is still somewhat low. While the implementation of the calculations in R is useful, it is not necessarily difficult or groundbreaking; nor do there appear to be any surprising results or new insights shown or described.

A quick glance at the following shows that they may be more appropriate places to publish for the current level of the manuscript:

* The Journal of Statistical Software

* The R Journal

These journals are specifically geared toward providing a place to publish these types of implementations. The Journal of Statistical Software arguably tends toward more sophisticated analyses than the R Journal.

Answer: We choose the PlosOne Journal to publish our study, due to scientific importance of this journal and coverage of readers around the world. In addition, before submit the manuscript to PlosOne, we searched and found similar articles presenting R packages and social behavior; both topics, R package and social behavior, are the focus of our manuscript. Our main objective in developing and making available the socialh package in R environment was to facilitate access to functional tools for researchers who have difficulty using software based on computational language to perform data analysis.

It still seems necessary to demonstrate some overall usefulness of this new package in at least two more situations.

An alternative would be some simulation studies using the package that could shed light on the capabilities or limitations of the methodology.

It would be quite simple to implement bootstrap statistical tests to produce confidence limits and/or tests of point hypotheses.

To summarize:

* The package implementation is useful, but on its own is more suited to another journal setting.

* One example of an analysis is interesting but does not seem to yield any insight or new results.

* Perhaps performing two or more analyses could allow the authors to draw some conclusion about the methodology.

* Perhaps using simulation studies the authors could derive some conclusion about the methodology.

* It would be easy to implement some simple statistical methods --- maybe these would produce some interesting results for either this data set or others.

* Perhaps there is deeper statistical theory already extant that could be incorporated.

Answer: Ln 342 – 373: To implement the above suggestions, we determined the dominance index from the different methodologies available in R packages (David Score, Elo Rating, and I&SI) for the same data base (from Nellore cattle) used in the examples. Also, we performed an correlation analysis among the results obtained by all methods, including the Kondo and Hurnik index values from the socialh package.

A few minor comments:

* Line 345-346 Again, these are simply not large databases, especially for the simple calculations performed by the software.

Answer: Done

* Understanding that the parameters are already defined for the functions, it is still best to make them user-definable in general. This can be easily accomplished using default values, so the user never needs to set them if that is preferred.

Answer: We understand your concern. However, we applied the time-interval 10 seconds as an example value in the replacement() function, and we describe in the text that this value can be change by researcher according com the objectives on each study.
